# Serum 25-hydroxyvitamin D and risk of type 2 diabetes in older adults: A dose–response meta-analysis of prospective cohort studies: Retraction

**DOI:** 10.1097/MD.0000000000009788

**Published:** 2018-01-26

This is a retraction notification for the article, “Serum 25-hydroxyvitamin D and risk of type 2 diabetes in older adults: A dose–response meta-analysis of prospective cohort studies”^[1]^, which appeared in Volume 97, Issue 1 of *Medicine*. After publication, a reader brought to the journal's attention that there may be plagiarism in another published article. After investigation, it was clear that the authors had plagiarized multiple articles without giving attribution to them.
